# The Prognostic Effect of Serum Albumin Level on Outcomes of Hospitalized COVID-19 Patients

**DOI:** 10.1155/2021/9963274

**Published:** 2021-06-09

**Authors:** Yazan Abdeen, Ahmad Kaako, Zaid Ahmad Amin, Ala Muhanna, Luise Josefine Froessl, Mohammad Alnabulsi, Amira Okeh, Richard A. Miller

**Affiliations:** ^1^Pulmonary and Sleep Physicians of Houston,PA, Webster, TX, USA; ^2^Mercy Hospital Fort Smith, Fort Smith, AR, USA; ^3^Saint Michael's Medical Center, Newark, NJ, USA; ^4^Aix-Marseille University, Marseille, France; ^5^University of Illinois, Chicago, IL, USA

## Abstract

As SARS-CoV-2 continues to spread across the globe and significantly impacts health-care systems and strains resources, identifying prognostic factors to predict clinical outcome remains essential. We conducted a retrospective cohort study to further explore the prognostic value of serum hypoalbuminemia and other factors in hospitalized COVID-19 patients. The primary endpoint was defined as the risk of in-hospital mortality. 300 patients were included in the analysis, with 56% being male and a mean (±SD) age of 61.5 ± 15.3 years. The mean (±SD) albumin was 2.86 ± 0.5 g/dL. Our analysis showed that patients with in-hospital mortality had lower albumin levels than patients without in-hospital mortality (2.6 ± 0.49 vs. 2.9 ± 0.48 g/dL, respectively, with *P* value = <0.001). A multivariant logistic regression analysis was subsequently conducted, and after adjustment, the serum albumin level remained a strong predictor of the primary outcome. Based on the data gathered, we were able to create a model predictive of mortality in this patient group based on the serum albumin level and other pertinent factors. In this model, with all other variables remaining constant, each one-unit increase in albumin is estimated to reduce the odds of mortality by 73%. Our results strengthen the current available data on the prognostic value of serum albumin in COVID-19 patients and provide a model to predict in-hospital mortality.

## 1. Introduction

In December 2019, SARS-CoV-2 (Severe Acute Respiratory Syndrome Coronavirus 2) was first identified after a cluster of viral pneumonia cases were detected in Wuhan (Hubei province, China). The virus has rapidly spread across the globe to be characterized a world-wide pandemic by the WHO on March 11, 2020. As of December 5, 2020, there have been over 66 million reported cases of SARS-CoV-2 infection globally and over 1.5 million deaths [1].

The pandemic has had wide-spread effects on the global and US health-care systems with significant strain on available resources. According to the US CDC COVID-19 surveillance, as of the week ending on November 28, 2020, weekly hospitalization rates were at their highest level since the beginning of the pandemic, with a cumulative COVID-19-associated hospitalization rate of 262.8 per 100,000 population [2]. The HHS recently released granular data from 2200 counties across the US detailing hospital and ICU usage. The data shows that, in 126 counties, hospitals are at least 90% occupied. On a national level, almost 15% of inpatient beds are currently estimated to be occupied by a COVID-19 patient. Moreover, since the beginning of the pandemic, studies have shown a significant increase in mortality related to non-COVID-related illnesses [3]. These facts highlight the pandemic's wide-reaching impact that extends beyond the direct mortality related to SARS-CoV-2 itself. Some current data suggest that case-fatality rates are trending down independent of confounding factors [4]. The data support the idea that improved treatment strategies and a better understanding of prognostic factors may reduce case fatality. However, clear markers that predict an adverse clinical outcome early on and identify patients that are potential candidates for select current treatment options remain elusive. Previous studies have examined the characteristics of patients with severe COVID-19.

Some commonly identified factors include male gender, age, comorbidities, especially hypertension, and serum markers such as an elevated D-dimer level, CRP, or LDH [5–8]. Some studies suggest that serum albumin levels may also be associated with prognosis in hospitalized patients with COVID-19 [9].

Therefore, we conducted this study to further evaluate the significance of serum albumin levels on admission and other factors concerning morbidity and mortality of hospitalized patients with COVID-19.

## 2. Materials and Methods

### 2.1. The Study Design and Oversight

We have conducted a retrospective cohort study based on a comprehensive review of Electronic Medical Records (EMRs). The approval of the Institutional Review Board (IRB) (#40/21) was issued before data collection. The study variables were extracted from the EPIC system at Saint Michael's Medical Center (SMMC) and were added directly into and stored in a password-protected EXCEL worksheet accessible only to the study investigators. No paper files were printed or stored. All the data were kept private and confidential per IRB and HIPAA policies. The patients' informed consent requirement was waived by the IRB, based on the study's retrospective nature.

### 2.2. The Study Population

Saint Michael's Medical Center (SMMC) is an inner-city community hospital in Newark, New Jersey, with the New York Medical College affiliation. It serves a region with Latino and African American majority. SMMC encountered a high number of patients with COVID-19 during the pandemic. The EMRs were screened for all the adult patients (age ≥18 years) and admitted to Saint Michael's Medical Center (SMMC) with respiratory distress and confirmed COVID-19 between February 1, 2020, and April 30, 2020.

### 2.3. Study Outcomes

The primary outcome was in-hospital mortality. Our primary goal was to assess if the albumin level on admission has any prognostic value for mortality among patients presented and admitted with confirmed COVID-19. Our secondary goal was to evaluate any other potential prognostic variables that may predict outcomes associated with COVID-19.

## 3. Definitions

For analysis classification, a fatal case of COVID-19 was defined as any death that occurred during the patient's hospitalization with a confirmed diagnosis of COVID-19 by PCR testing collected through a nasopharyngeal swap. A nonfatal case was defined as a confirmed case of COVID-19 by PCR in a hospitalized patient who had not died (whether discharged or still hospitalized) as of April 30, 2020

### 3.1. Inclusion Criteria

The main inclusion criteria were the following:

1. Adult patients (age ≥18 years) presenting with active respiratory symptoms related to SARS-CoV-2 infection, including shortness of breath, cough, and oxygen saturation less than 92% on room air
2. Confirmed case of COVID-19 by PCR
3. Patients having serum albumin level drawn on the day of admission

To minimize confounding factors that may affect our evaluation of albumin's effect on mortality, we excluded patients with a known history of End-Stage Liver Disease (ESLD), liver cirrhosis, or nephrotic syndrome on presentation since these medical conditions may independently alter the albumin levels [10].

## 4. Methods

Electronic medical records (EMR) were reviewed to extract the study variables, which included demographics (age, sex, and race), clinical variables (date of admission to the ICU, BMI, and cardiac ejection fraction), laboratory variables that include admission day blood workup (serum albumin level, ferritin, lymphocytes, CRP, lactic acid, and GFR), and comorbidities including Acute Kidney Injury (AKI), Diabetes Mellitus (DM), Chronic Obstructive Pulmonary Disease (COPD), Congestive Heart Failure (CHF), and Atrial Fibrillation (AF) ESLD and ESRD. A total of 302 patients were identified to be admitted with respiratory distress and positive COVID-19 in the defined period. Three hundred patients were included in the final analysis after excluding two patients with a history of ESLD. None of the patients had a documented a history of nephrotic syndrome. Among the included subjects was one patient with HIV on antiretroviral therapy and a normal CD4 count, as well as four patients with a history of malignancy not actively on chemotherapy and without neutropenia on admission.

The primary outcome was in-hospital mortality for all subjects. To evaluate the effect of some continuous variables on the outcome of interest, we classified the subjects into subgroups. All the patients were classified into two subgroups based on the BMI (BMI≥ 30 kg/m2 vs. <30 kg/m2). Furthermore, all the patients were classified into two subgroups based on the GFR (GFR ≥60 mL/min vs. GFR <60 mL/min).

## 5. Statistical Analysis

We described the cohort characteristics using frequency tables with proportions for binary and categorical variables and mean with standard deviation for the continuous variables. We reported the descriptive statistics for all the patients and then categorized them into two subgroups (patients with fatal cases and patients with nonfatal cases) based on the mortality outcome. Then, we analyzed the binary, categorical, and continuous variables between the two subgroups and measured the association between the two, using the Pearson *χ*2-test for binary and categorical variables and the *t*-test for continuous variables. We examined the association between the multiple variables and the binary outcome (in-hospital mortality) by conducting a multivariant (adjusted) logistic regression analysis. We did not include highly correlated variables within the regression analysis model to avoid collinearity. A *P* value < 0.05 is considered statistically significant. All calculations were made using STATA version 14.2 (StataCorp, College Station, TX, USA).

## 6. Results

A total of 302 patients were identified to be admitted with respiratory symptoms, including dyspnea, cough, and hypoxia related to a confirmed diagnosis of COVID-19 by PCR.

Three hundred patients were included in the final analysis after excluding two patients with a history of liver cirrhosis.

Table 1 shows the patients' demographic and clinical characteristics in the study and the differences between the two subgroups based on the mortality outcome. Overall, 59% of patients were men, and the mean (±SD) age was 61.5 ± 15.3 years. Most patients (86%) were African American and Hispanic, and the remainder is outlined in Table 1.

As expected, older patients have a higher risk of mortality with a higher mean age in the mortality subgroup (65 vs. 60 years, *P* value = 0.006). Notably, more men were admitted to the hospital with COVID-19 than women (59% vs. 41%), and almost three fourths (72%) of the patients with in-hospital mortality were men (*P* value = 0.003).

Half of the patients who are admitted had GFR <60 mL/min. Notably, there is a higher proportion of kidney insufficiency in patients with in-hospital mortality (68% vs. 43%, *P* value = <0.001), making the GFR category on admission a strong predictor of mortality. One-third (33%) of the patients were admitted to the ICU. As expected, ICU admission was a strong predictor for mortality with a remarkably higher proportion of ICU admission in the subgroup with in-hospital mortality (74% vs. 16%, respectively, with *P* value =<0.001). Higher rates of obesity are noted among patients admitted with COVID-19, with almost half (47%) of the patients having a BMI≥ 30 kg/m2. D-dimer, ferritin, and lactic acid levels were all more elevated in patients with in-hospital mortality compared to patients without in-hospital mortality. A statistically significant difference was found on univariate analysis as outlined in the table. There was no statistically significant difference between the two subgroups (patients with and without in-hospital mortality) in regards to race, BMI, obesity, DM, COPD, CHF, COPD, AF, and lymphocyte count.

The mean (±SD) albumin was 2.86 ± 0.5 g/dL. Patients with in-hospital mortality have lower albumin levels than patients without in-hospital mortality (2.6 ± 0.49 vs. 2.9 ± 0.48 g/dL, respectively, with *P* value = < 0.001), making albumin a potentially strong indicator for mortality in patients admitted with COVID-19 based on the univariant analysis.

**Figure 1:**
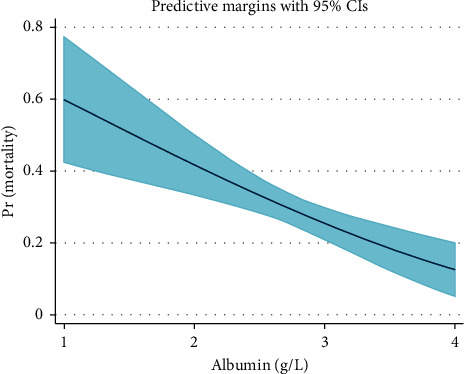
A predictive model to estimate the odds of mortality based on the albumin level on admission, including predictive margins with 95% confidence intervals.

Furthermore, we have examined the association between the multiple variables and the binary outcome (in-hospital mortality) by conducting multivariant (adjusted) logistic regression analysis as listed in Table 2. All variables were considered covariates in the multivariate model except for BMI and AKI, which were excluded due to collinearity with obesity and GFR category. Albumin level remained a strong predictor for the primary outcome (in-hospital mortality) after adjustment, as shown in Table 2. Finally, we performed a stepwise logistic regression analysis, with a conservative significance threshold of 0.05 to determine the qualification of data for entry into or deletion from the model, as shown in Table 3. All reported *P* values are nominal and two sided and were not adjusted for multiple comparisons. A predictive model was developed to estimate the odds of mortality based on the albumin level upon admission. In our predictive model, holding all the other variables constant, each one-unit increase in albumin is estimated to reduce the odds of mortality by 73%. The predictive margins are plotted in Figure 1, based on our predictive model with 95% confidence intervals.

## 7. Discussion

This retrospective cohort study was conducted to identify factors predictive of poor clinical outcome in hospitalized COVID-19 patients. COVID-19 has the unique characteristic of inducing severe disease in young, otherwise healthy patients [8]. Previous studies have identified some clinical risk factors. However, some of these elements are imperfect, and discovering more dynamic markers is imperative during this ongoing pandemic. Prognostication is essential to determine which patients will require more aggressive treatment early on in the disease course and for effective resource allocation in the context of the current increased burden on our health-care system.

Primarily, the results of our investigations showed a strong statistically significant association between hypoalbuminemia upon initial presentation and mortality in the group of patients included in our study. Albumin, a small globular protein, is the most abundant human plasma protein and accounts for more than two-thirds of plasma oncotic pressure. It is synthesized by the liver and serves many essential functions [11]. In particular, albumin transports various substances in the bloodstream, is a free oxygen radical scavenger, has antioxidant effects, and can even inhibit platelet aggregation. It plays a vital role in many processes, especially inflammation.

Inflammation has also been noted to play a central role in the SARS-CoV-2 infection. This virus can induce a wide variety of clinical expressions, ranging from mild flu-like symptoms to severe systemic disease with multiorgan failure and ARDS. The physiopathology behind the development of severe disease in some patients and milder disease in others is unclear and has yet to be more extensively characterized. However, the current literature supports the idea that clinical severity may be due to virally driven hyperinflammation [12]. In support of this theory, it has been shown that coronaviruses possess particular proteins that dysregulate the immune response in infected individuals, which can induce excessive inflammatory states [13]. The RISC-19-ICU (Risk Stratification of COVID-19 patients in the intensive-care unit) was developed in Europe to evaluate the characteristics of patients with a critical illness. In this cohort of patients, the factors identified to be associated with more severe disease (lactate, D-dimer, creatinine, and others) are markers of oxygenation deficit and renal and microvascular dysfunction, as well as coagulopathy, pathological processes associated with high levels of inflammation [6].

Previous research suggests that low serum albumin may be significantly correlated with increasing physiologic stress, such as, in particular, inflammation. During proinflammatory states, cytokines increase capillary permeability and allow nutrients, including albumin, to escape into the interstitium. Decreased albumin levels may also be secondary to the increased protein degradation by cells to provide amino acid building blocks for the interstitial matrix synthesis and cell proliferation. Therefore, the reduced serum albumin levels in inflammatory states may reflect the increased escape of albumin from the vascular space and uptake by cells rather than a decrease in synthesis [14].

A cross-sectional study found a correlation between higher inflammatory markers in the serum of patients with more severe COVID-19, including cytokines such as IL-2R, IL-6, and TNF-a, suggesting that the inflammation level may predict disease severity in COVID-19 [15].

Moreover, previous retrospective cohort studies conducted in China have found a significant relationship between hypoalbuminemia in COVID-19 patients and mortality [9, 16]. The association between serum hypoalbuminemia and poor clinical outcome detected in our study is, therefore, plausible and has a well-characterized biological background and is supported by previous findings.

Furthermore, the concept of a relationship between low serum albumin and more severe disease progression has previously been found to exist in ARDS independent of COVID-19. Retrospective cohort studies have found statistically significant associations between both mortality and length of mechanical ventilation and low serum albumin levels in patients with ARDS [17], [10]. Similarly, a prospective study of 424 patients with community-acquired pneumonia found an independent association between low serum albumin levels and 28-day mortality [18]. The inverse relationship between serum albumin levels and survival has been established in many other clinical circumstances, such as end-stage renal disease and preoperative context [19, 20]. Additional studies have shown that hypoalbuminemia may even be a valuable tool in monitoring the progression and severity of ARDS, superior to CRP or LDH levels [21].

Our study has not only demonstrated a significant correlation between serum albumin on admission and mortality among patients hospitalized with COVID-19 but also developed a predictive model that estimated the probability of mortality based on the serum albumin level on admission, and other significant factors are shown in Table 3 and Figure 1.

Furthermore, our results identified several other factors that showed a significant association with inpatient mortality. Not surprisingly, age and ICU admission were predictive of mortality. Both male gender and impaired kidney function with a GFR of <60 were independent factors of mortality in our predictive model. These observations are in line with previous retrospective studies that have found clinical factors such as male gender, increasing age, and underlying conditions, such as CKD, to be predictive of poorer outcomes [7, 8, 22].

Our study has its limitations. It is a retrospective and single-center study which produces its inherited caveats. Conducting prospective, multicenter studies would lead to more robust evidence to the link of hypoalbuminemia and mortality. Moreover, our study was conducted in a center that serves geographic areas with higher Hispanic and African American populations, precluding generalization nationally or globally. However, given the correlation of our findings with previous studies conducted in China, it appears that our results are in line with the broader population of COVID-19 patients.

Based on the correlation between hypoalbuminemia and mortality, it is intriguing to investigate the value of therapeutic albumin infusions in hospitalized COVID-19 patients with severe disease. Therapeutic albumin infusions have previously been compared to crystalloid in patients with non-COVID-19-related ARDS. Although it did not reduce mortality, however, improved oxygenation was suggested [23]. Unfortunately, there is currently a lack of robust data on albumin infusion's therapeutic value and even more scarce data in patients with COVID-19. The observed hypoalbuminemia could be merely an expression of an underlying inflammatory state rather than a direct cause of morbidity and mortality.

In conclusion, serum albumin level was shown to have a significant association with mortality in our study population and could be considered a strong predictor for mortality in hospitalized patients with COVID-19. Based on our predictive model, it is possible to estimate the odds of mortality based on the serum albumin level on admission. Both the physiopathology of the COVID-19 process and previous evidence support the validity of our data. The investigation of the benefit of therapeutic albumin infusions in this group of patients may be of interest in the future.

## Figures and Tables

**Table 1:** Case status and demographic and clinical characteristics of patients with COVID-19, with and without in-hospital mortality.

Variable	All patients	Patients with in-hospital mortality	Patients without in-hospital mortality	*P* value
Age mean (sd), yr	61.5 (15.3)	65.3 (16.11)	59.9 (15.4)	**0.006**
Male	59.7 (14.4)	63.1 (14.4)	57.9 (14.2)	
Female	64 (16.2)	70.9 (12.4)	62.3 (16.6)	

*Sex, no./total no. (%)*				
Male	178/300 (59%)	63/87 (72%)	115/213 (53%)	**0.003**
Female	122/300 (41%)	24/87 (28%)	98/213 (46%)	

*Race, no./total no. (%)*				
African American	133/300 (44%)	37/87(43%)	96/213(45%)	0.687
Hispanic	125/300 (42%)	36/87(41%)	89/213 (42%)	
White	21/300 (7%)	7/87 (8%)	14/213 (7%)	
Other	21/300 (7%)	7/87(8%)	14/213 (7%)	

BMI, mean (SD) (kg/m^2^)	30.9 (7.8)	30.5 (7.1)	31 (8)	0.749
Obesity (BMI ≥30)	140/300(47%)	41/87 (47%)	99/213(46%)	0.919
GFR (<60)	151/300 (50%)	59/87 (68%)	92/213(43%)	**0.001**
AKI	124/300 (41%)	55/87 (63%)	69/213 (32%)	**0.001**
DM	125/300 (42%)	42/87 (49%)	83/213 (39%)	0.138
HTN	190/300 (63%)	52/87 (60%)	138/213 (65%)	.0413
CAD	32/300 (11%)	10/87 (11%)	22/213 (10%)	0.767
COPD	25/300 (6.2%)	5/87 (6%)	20/213 (9%)	0.300
AF	15/300 (5%)	4/87 (5%)	11/213 (5%)	0.838
CHF	37/300(12%)	21/213 (10%)	16/87(18%)	**0.041**
ICU admission	99/300 (33%)	64/87 (74%)	35/213 (16%)	**0.001**
Albumin, mean (SD)	2.86(0.5)	2.6 (0.49)	2.9(0.48)	**0.001**
CRP	10.7 (8.3)	8.8 (6.5)	15.7 (10)	**0.001**
Lymphocytes	0.95 (0.5)	0.89 (0.5)	0.97 (0.5)	0.183
D-dimer (Mic/L)	4237 (12623)	3120 (8835)	7036 (188850)	**0.024**
Ferritin	1269 (2739)	852 (846)	2280 (4767)	**0.001**
Lactic acid	2.1 (1.8)	1.9 (1.7)	2.4 (2)	**0.046**

BMI: body mass index, GFR: glomerular filtration rate, AKI: acute kidney injury, DM: diabetes mellitus, HTN: hypertension, CAD: coronary artery disease, COPD: chronic obstructive pulmonary disease, AF: atrial fibrillation, CHF: congestive heart failure, CRP: C-reactive protein.

**Table 2:** Multivariate associations with in-hospital mortality among patients hospitalized with COVID-19.

Factor	Odds ratio (95% CI)	*P* value
Age	1.07 (1.03–1.1)	**0.001**
Sex	0.31 (0.12–0.78)	**0.013**
Race	0.80 (0.50–1.28)	0.358
Obesity (BMI ≥30)	1.54 (0.69–3.48)	0.291
GFR (<60)	2.65 (1.03–6.79)	0.042
DM	0.44 (0.18–1.34)	0.090
HTN	1.14 (0.41–3.14)	0.793
CAD	0.62 (0.16–2.40)	0.488
COPD	1.07 (0.24–4.71)	0.924
AF	0.82 (0.14–4.72)	0.827
CHF	1.01 (0.61–5.44)	0.281
ICU admission	42.52 (14.85–121.74)	**0.001**
Albumin, mean (SD)	0.35 (0.139–0.89)	**0.028**
CRP	1.05 (0.99–1.11)	0.072
Ferritin	1.00 (0.99–1.00)	0.090
D-dimer	0.99 (0.99–1.00)	0.300
Lymphocyte	1.53 (0.63–3.64)	0.340

BMI: body mass index, GFR: glomerular filtration rate, AKI: acute kidney injury, DM: diabetes mellitus, HTN: hypertension, CAD: coronary artery disease, COPD: chronic obstructive pulmonary disease, AF: atrial fibrillation, CHF: congestive heart failure, CRP: C-reactive protein.

**Table 3:** Factors associated with in-hospital mortality among patients hospitalized with COVID-19 in the final multivariate model.

Factor** **	Odds ratio (95% CI)	*P* value
Age** **	1.07 (1.03–1.09)	**<0.001**
Sex** **	0.24 (0.11–0.52)	**<0.001**
GFR (<60)** **	2.16 (1.01–4.63)	**0.048**
ICU admission** **	31.24 (13.48–72.34)	**<0.001**
Albumin, mean (SD)** **	0.27 (0.13–0.54)	**<0.001**

## Data Availability

Data results are demonstrated in the result section and further explained in the discussion section. In addition, Table 1, Table 2, and Figure 1 demonstrate the results.
